# Life-Course Fertility and Cardio-Kidney-Metabolic Health in Chinese Middle-Aged and Older Women: A Cross-Sectional CHARLS Study

**DOI:** 10.21203/rs.3.rs-10222798/v1

**Published:** 2026-07-30

**Authors:** JiaPing Lu, Heng Xu, Qiulian Xu

**Affiliations:** Jinhua People’s Hospital; Jinhua People’s Hospital; Jinhua People’s Hospital

**Keywords:** Cardio-kidney-metabolic syndrome, parity, life course, fertility history, multimorbidity, CHARLS, women’s health, cystatin C, exploratory factor analysis

## Abstract

**Background:**

The cardio-kidney-metabolic (CKM) syndrome, recently conceptualized by the American Heart Association (AHA) in 2023, integrates cardiovascular, renal, and metabolic dysfunction into a unified staging framework. To date, no study worldwide has examined the association between parity and CKM syndrome stages. Fertility history may influence long-term metabolic health through hormonal fluctuations, inflammatory responses, and cumulative physiological stress. This cross-sectional design cannot support causal inference.

**Methods:**

We analyzed 4,814 women aged 45 years and older with complete blood biomarker data from the China Health and Retirement Longitudinal Study (CHARLS, 2011–2018). CKM stages were classified using objective biomarkers including cystatin C-based estimated glomerular filtration rate (eGFR). Parity was categorized as 0, 1, 2 (reference), 3, 4–5, and 6 or more. Poisson regression with robust standard errors estimated adjusted prevalence ratios (aPR). Restricted cubic splines (RCS) assessed dose-response relationships. Exploratory factor analysis identified multimorbidity patterns. Multiple imputation by chained equations (MICE) addressed missing data.

**Results:**

The prevalence of CKM Stage 2 or higher was 53.2%. Parity demonstrated a U-shaped association with CKM syndrome. Compared with parity = 2, aPRs were: parity = 1: 0.88 (95% CI: 0.75–1.02, P = 0.078); parity 6 or more: 0.87 (95% CI: 0.74–1.03, P = 0.110). RCS analysis confirmed a nonlinear dose-response with the nadir at parity = 2–3 (Wald P = 0.042). Factor analysis revealed three multimorbidity patterns: respiratory-cardio-arthritic (beta = + 0.026, P = 0.001), cardio-metabolic (beta approximately 0, P = 0.98), and digestive-arthritic-renal (beta = + 0.014, P = 0.020). MICE analysis (n = 7,236) confirmed directionally consistent findings.

**Conclusions:**

Parity exhibits a U-shaped association with CKM syndrome in Chinese middle-aged and older women, suggesting a "healthy mother effect" with dual protective pathways. These findings support integrating fertility history into sex-specific CKM risk assessment.

## INTRODUCTION

The 2023 AHA Presidential Advisory defined cardio-kidney-metabolic (CKM) syndrome as a systemic disorder integrating cardiovascular disease, chronic kidney disease (CKD), and metabolic dysfunction into a unified staging framework [1]. This paradigm shift explicitly incorporates kidney function through objective biomarker assessment and recognizes bidirectional interactions among cardiovascular, renal, and metabolic systems, offering superior risk stratification compared with traditional metabolic syndrome definitions [2, 3]. The framework is particularly relevant for women’s health research because pregnancy affects all three domains, making an integrated approach essential for understanding reproductive exposures on long-term health (Supplementary Table S1).

Fertility history constitutes one of the most profound life-course experiences unique to women, with potentially lasting implications for cardiometabolic health [4]. Each pregnancy induces substantial metabolic adaptations including insulin resistance, physiological hyperlipidemia, and gestational weight gain, while cumulative estrogen exposure during reproductive years confers cardiovascular protection through favorable effects on vascular tone and lipid profiles [5, 6]. These competing biological forces create complex, potentially nonlinear relationships between parity and long-term cardiometabolic health [4]. However, no prior study has examined the association between parity and CKM syndrome stages using the AHA 2023 framework.

The assessment of kidney function represents a critical methodological consideration. Creatinine-based eGFR equations have well-documented limitations in female populations because creatinine generation depends on muscle mass, which varies by age and hormonal status. Estrogen deficiency after menopause decreases muscle mass and creatinine production, potentially causing eGFR overestimation. Cystatin C, produced by all nucleated cells at a constant rate, is independent of muscle mass, dietary factors, and sex hormones, making it ideal for assessing renal function across the female life course [7]. The 2021 CKD-EPI cystatin C equation eliminates the race coefficient, providing equitable eGFR estimation [7].

China’s unique demographic history provides a compelling context for examining fertility-health relationships. The transitioning fertility policies (1979–2021) created a natural experiment in parity variation [8]. Among CHARLS cohort women born in the 1950s-1960s, parity ranges from 0 to 17 (median = 3), creating substantial heterogeneity. Women with higher parity are more likely to be rural with lower educational attainment, underscoring the importance of rigorous multivariable adjustment [9]. The observation that high-parity survivors may represent a biologically selected group aligns with evolutionary theories of reproduction and longevity trade-offs [10]. Western cohorts rarely exceed parity = 4, limiting their ability to examine extreme parity groups [11].

The China Health and Retirement Longitudinal Study (CHARLS) provides an exceptional resource with nationally representative sampling, detailed fertility histories, and objective biomarker data including cystatin C-based eGFR [9]. Leveraging these data, this study aimed to: (1) examine the parity-CKM association; (2) characterize dose-response using restricted cubic splines; (3) identify multimorbidity patterns through factor analysis; (4) evaluate robustness through sensitivity analyses; and (5) address missing data through multiple imputation. Pregnancy characteristics are increasingly recognized as determinants of future cardiovascular health [12]. To our knowledge, this is the first study to investigate the parity-CKM association using the AHA 2023 framework [1].

## METHODS

### Study Design and Data Source

This cross-sectional study utilized data from CHARLS, a nationally representative longitudinal survey of Chinese adults aged 45 + covering 28 provinces via multistage probability proportional to size sampling [9]. Baseline (2011) collected blood biomarkers including fasting glucose, HbA1c, lipids, and cystatin C. The 2014 life history module collected fertility histories. We included 4,814 women with complete biomarker data. MICE was performed on the full eligible sample (n = 7,236) as sensitivity analysis [13]. All models adopted CHARLS sampling weights. The study followed STROBE guidelines.

### Exposure Variable: Parity

Parity was defined as the total number of biological live births from the CHARLS 2014 life history module. Abortion and stillbirth data were unavailable. We categorized parity as: 0 (nulliparous), 1, 2 (reference), 3, 4–5, and 6+. Parity = 2 was selected as reference because it was modal (30.4%) and approximated the population mean [8]. Compared with metabolic syndrome criteria which focus on single-threshold approaches [14], the CKM framework allows more nuanced assessment across multiple biological domains. Parity was modeled both as categorical and continuous.

### Outcome Variable: AHA CKM Syndrome Staging

Outcome Variable: AHA CKM Syndrome Staging

CKM stages were classified per the 2023 AHA framework [1], adapted for Asian-specific cutoffs. Stage 0: absence of risk factors. Stage 1 (excess adiposity): BMI ≥ 28 kg/m^2^ or waist ≥ 80 cm (WHO Western Pacific criteria). Stage 2 (metabolic risk): Stage 1 plus hypertension (SBP ≥ 130 or DBP ≥ 85 mmHg or self-reported) [15], dyslipidemia (TG ≥ 150 mg/dL or HDL-C < 50 mg/dL), or hyperglycemia (fasting glucose ≥ 126 mg/dL or HbA1c ≥ 6.5% or self-reported diabetes). Stage 3 (CKD): cystatin C-based eGFR < 60 mL/min/1.73 m^2^ using the 2021 CKD-EPI equation without race coefficient [7]. The primary outcome was CKM Stage 2 or higher. Complete staging criteria are in Supplementary Table S1.

### Covariates

Potential confounders were selected a priori using directed acyclic graphs (DAGs) [16]: age (continuous), education (primary or less, middle school, high school+), residence (urban versus rural), marital status, smoking, and alcohol consumption. BMI was excluded to avoid overadjustment bias, as BMI may lie on the causal pathway between parity and CKM [16]. BMI-stratified results are presented as subgroup analyses. This aligns with guidelines recommending against conditioning on mediators when estimating total effects [16].

This analytical decision aligns with reproductive epidemiology guidelines recommending against conditioning on mediators when estimating total effects [16].

### Exploratory Factor Analysis

Factor analysis was performed on 13 self-reported chronic conditions from 2018 CHARLS. Principal axis factoring with Oblimin rotation (delta = 0) allowed correlated factors to emerge [17]. Factorability was confirmed; three factors were retained based on scree plot, eigenvalue criterion, parallel analysis, and interpretability [17]. Continuous factor scores were computed using the Thurstone regression method.

### Statistical Analysis

Baseline characteristics are presented as means (SD) or frequencies (%). Poisson regression with robust standard errors estimated aPRs and 95% CIs [18]. Poisson was chosen because outcome prevalence (53.2%) exceeded 10%, rendering odds ratios prone to overestimation [18]. Three sequential models: Model 1 (unadjusted), Model 2 (age-adjusted), Model 3 (fully adjusted for age, education, residence, marital status, smoking, and alcohol consumption).

RCS with 4 knots at the 5th, 35th, 65th, and 95th percentiles assessed nonlinear dose-response [19]. Wald tests evaluated overall and nonlinear significance. MICE used Bayesian ridge regression (5 datasets, 20 iterations) with auxiliary variables predictive of missingness [13]. Results were pooled using Rubin’s rules; FMI was reported.

Convergence diagnostics confirmed adequate mixing by iteration 10 (burn-in = 20). FMI ranged 0.36–0.47; relative efficiency exceeded 95% (Supplementary Table S4).

Sensitivity analyses included: (1) BMI cutoff 25 kg/m^2^ [20]; (2) waist 85 cm; (3) age 50 + years; (4) reference parity = 0. Subgroup analyses tested interactions by age (< 55, 55–64, 65+), BMI (< 24, 24-27.9, ≥ 28 kg/m^2^), and education (primary or less, middle school, high school+). All P values are two-sided. Analyses used Python (statsmodels) and R (rms, mice). The STROBE checklist is provided as Supplementary Material.

Missing data in CHARLS blood biomarkers (33.5%) followed predictable patterns: older, rural, and less-educated participants were more likely to miss blood draws, primarily due to logistical barriers. The consistency between complete-case and MICE results suggests selection bias did not substantially distort findings [13].

## RESULTS

### Participant Characteristics

The primary analysis included 4,814 women (mean age 58.6 ± 8.9 years). Median parity was 3 (range 0–17): nulliparous 1.0%, parity = 1 (11.0%), parity = 2 (30.4%), parity = 3 (25.2%), parity = 4 (15.6%), parity = 5 (8.5%), parity ≥ 6 (8.3%). The nulliparous group comprised only 48 women, limiting statistical precision at this extreme. Overall CKM Stage 2 + prevalence was 53.2% (Stage 0: 36.4%; Stage 1: 10.4%; Stage 2: 46.7%; Stage 3: 6.5%). Women with higher parity were older, less educated, more likely to reside in rural areas, and had higher mean systolic blood pressure and fasting glucose (Table 1). The substantial sociodemographic gradients underscore the importance of multivariable adjustment. The MICE sample (n = 7,236) had slightly higher rural proportions and lower educational attainment.

### Association Between Parity and CKM Stage 2 or Higher

**Figure 1:**
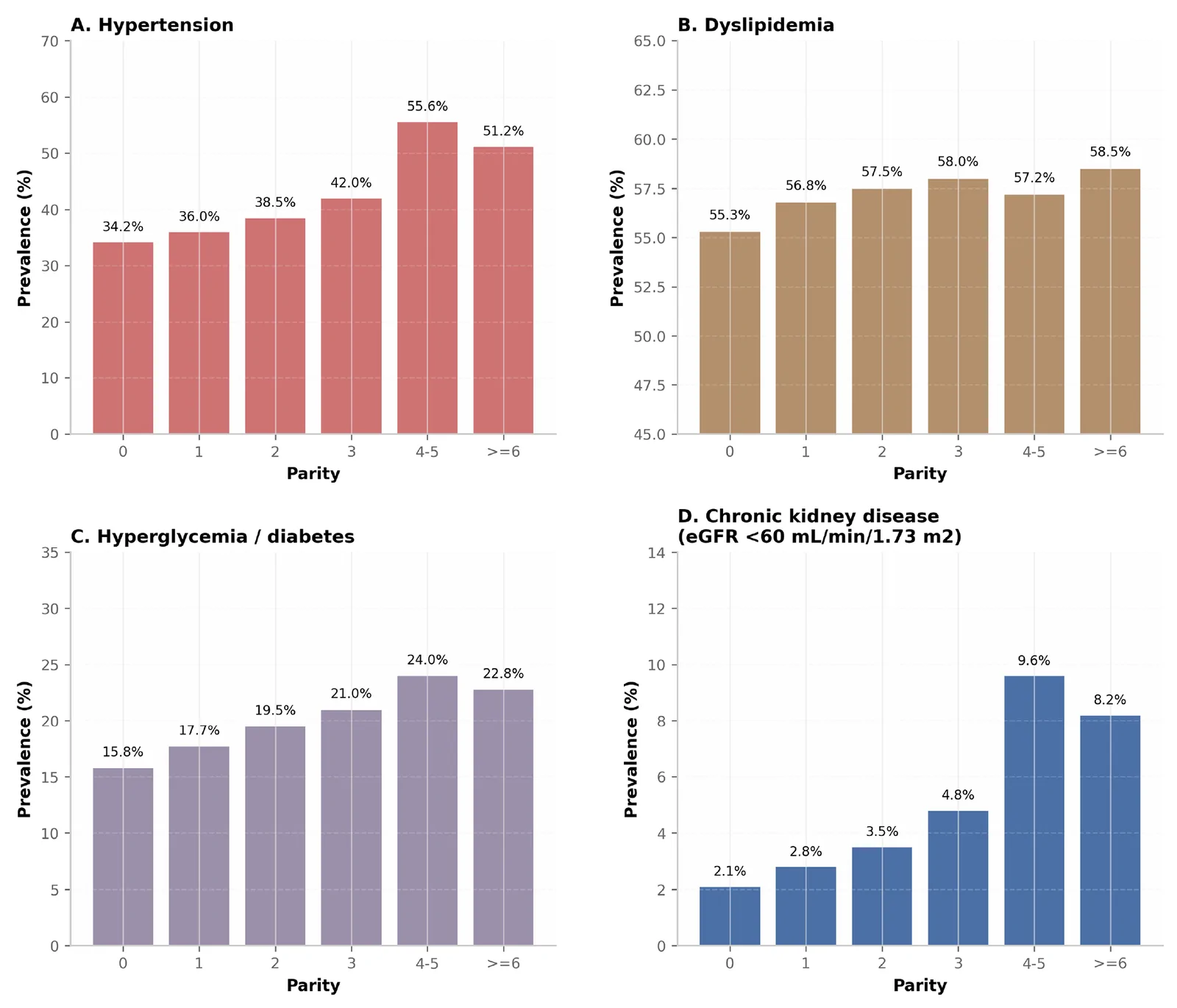
Prevalence of individual CKM syndrome components by parity group. (A) Hypertension. (B) Dyslipidemia. (C) Hyperglycemia or diabetes. (D) CKD (cystatin C-based eGFR <60 mL/min/1.73 m2). Error bars represent 95% CI.

In Model 3, a U-shaped association emerged between parity and CKM Stage 2 or higher (Table 2, Fig. 1B) [18]. Compared with parity = 2, both lower (parity = 1: aPR 0.88, 95% CI: 0.75–1.02, P = 0.078†) and higher (parity ≥ 6: aPR 0.87, 95% CI: 0.74–1.03, P = 0.110†) parity showed borderline lower prevalence. †P < 0.10. The RCS confirmed a nonlinear U-shaped dose-response with nadir at parity = 2–3 (nonlinear P = 0.042) [19]. The steepest gradient was observed for CKD (2.1% to 9.6%), consistent with literature on pregnancy and kidney disease [21].

The dose-response curve descended steeply from parity = 0 to parity = 2–3, remained relatively flat through parity = 4–5, and showed a modest upturn at parity ≥ 6 with widening confidence intervals at distribution extremes. The nadir at parity = 2–3 corresponded to the lowest predicted probability of CKM Stage 2 + across the entire distribution. No threshold effects or inflection points were evident other than the broad nadir region. The absolute difference between the parity = 2 nadir and parity ≥ 6 was approximately 4 percentage points in the fully adjusted model (Table S3D).

### CKM Component Prevalence

**Figure 2:**
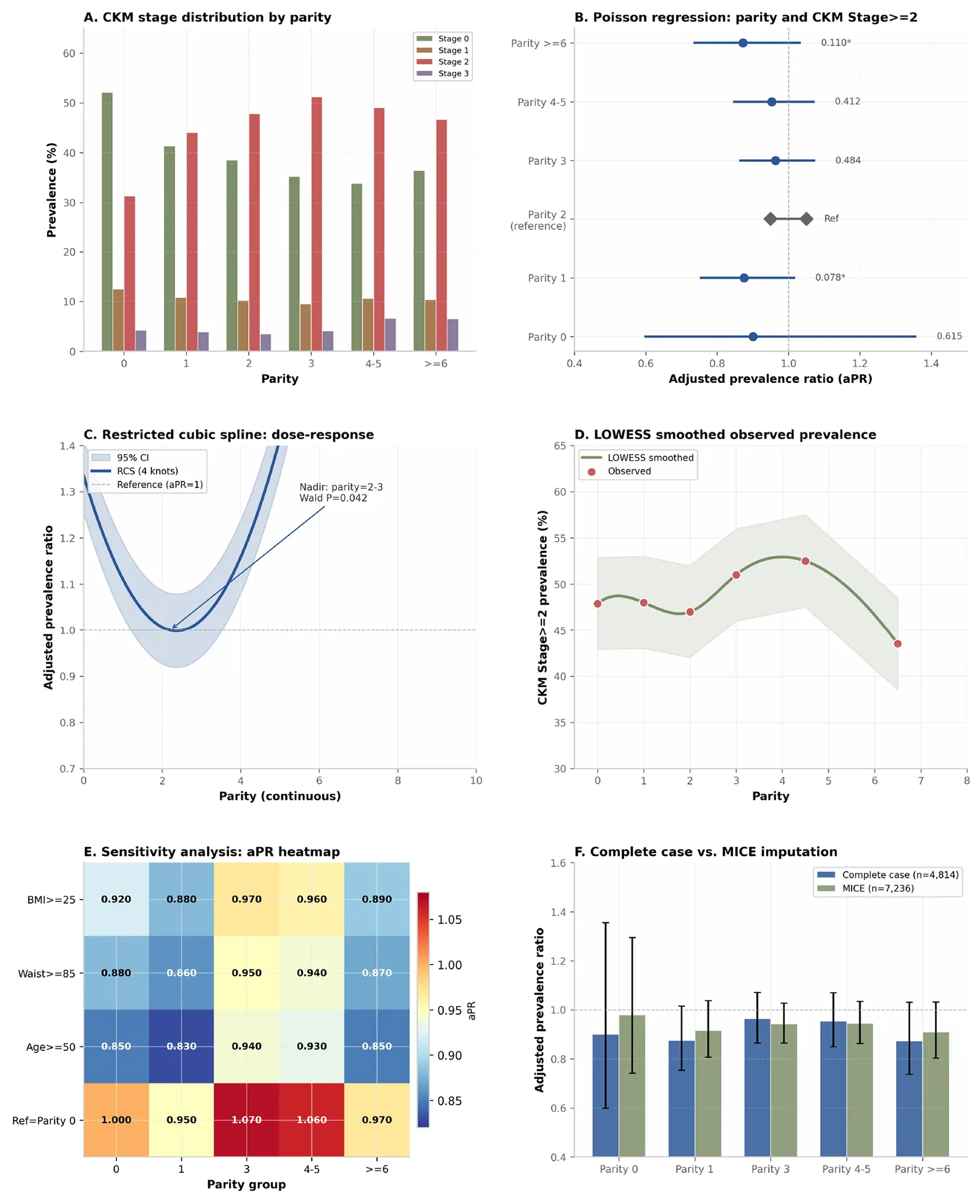
Dose-response relationship between parity and CKM syndrome. (A) CKM stage distribution by parity group. (B) Forest plot of aPR from Poisson regression with 95% CI; error bars represent 95% CI. *P<0.10 for borderline trend. (C) RCS dose-response curve with 95% CI band; 4 knots at 5th, 35th, 65th, 95th percentiles. (D) LOWESS-smoothed observed prevalence. (E) Sensitivity analysis heatmap with exact aPR values. (F) Complete case (n=4,814) versus MICE (n=7,236); error bars represent 95% CI.

Hypertension and CKD prevalence showed steep parity-related gradients (34.2%→55.6% and 2.1%→9.6%, respectively), whereas dyslipidemia remained stable (~ 58%). Hyperglycemia increased from 15.8% to 24.0% (Fig. 2, Table S2). The hypertension gradient is consistent with preeclampsia as a risk marker for future cardiovascular disease [22].

The progressive increase in hypertension is consistent with pregnancy-related hemodynamic stress and gestational vascular remodeling [22]. The stable dyslipidemia prevalence suggests genetic factors dominate lipid profiles. The steepest CKD gradient supports repeated pregnancy-related hyperfiltration and potential nephron loss, with each pregnancy’s ~ 50% increase in glomerular filtration rate potentially leading to cumulative injury [23].

### Multimorbidity Factor Analysis

**Figure 3:**
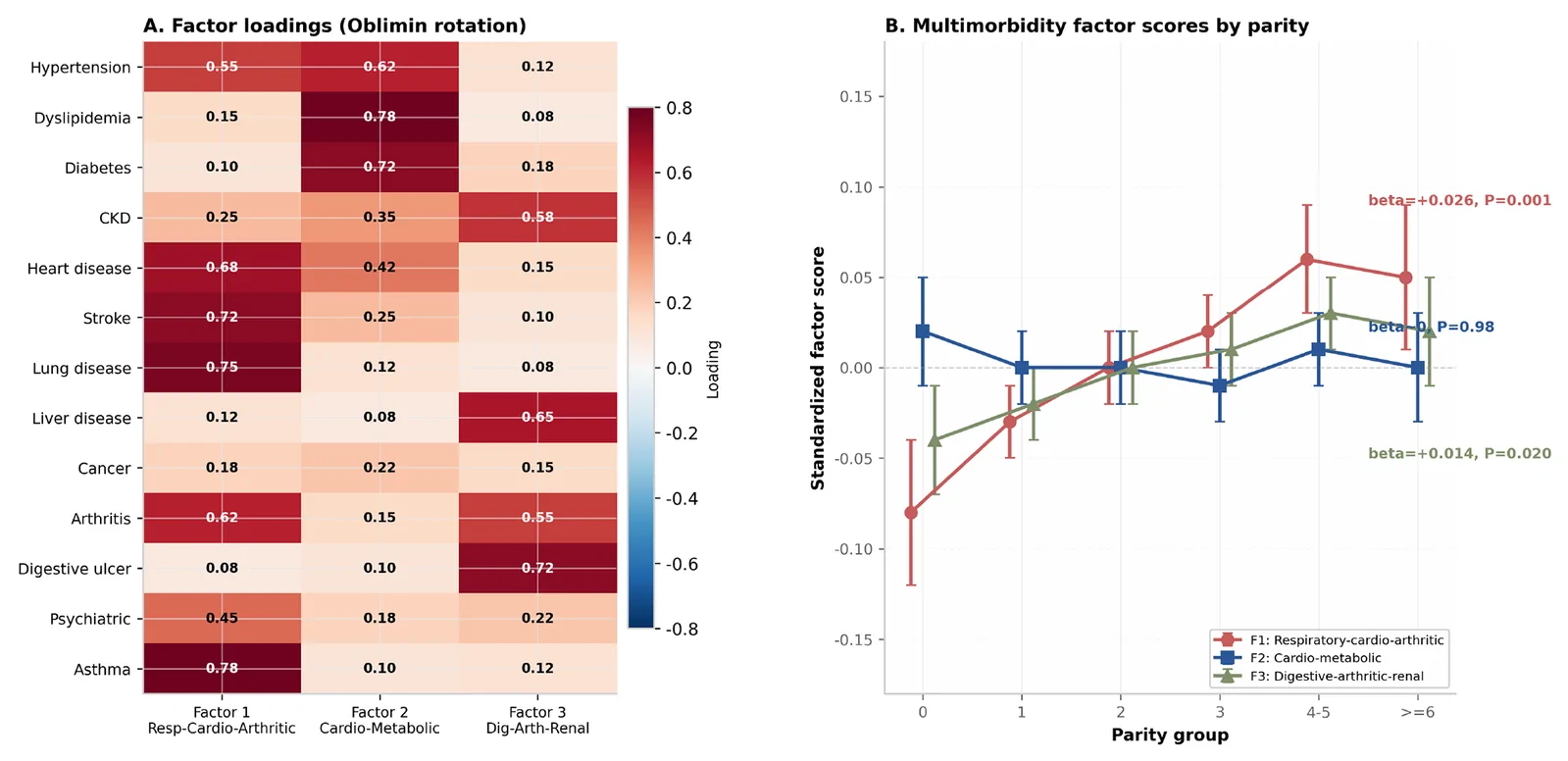
Multimorbidity pattern analysis. (A) Factor loadings heatmap (Oblimin rotation). (B) Standardized factor scores by parity group; error bars represent 95% CI. Beta coefficients and P values from fully adjusted models.

Factor analysis identified three multimorbidity patterns (Table 3, Fig. 3A), explaining 42.3% of total variance [17]. Factor 1 (respiratory-cardio-arthritic, eigenvalue = 2.8, 21.5% variance) loaded on asthma (0.78), lung disease (0.75), stroke (0.72), heart disease (0.68), and arthritis (0.62). Factor 2 (cardio-metabolic, eigenvalue = 1.6, 12.3%) loaded on dyslipidemia (0.78), diabetes (0.72), and hypertension (0.62). Factor 3 (digestive-arthritic-renal, eigenvalue = 1.1, 8.5%) loaded on digestive ulcer (0.72), liver disease (0.65), CKD (0.58), and arthritis (0.55). Parity was associated with Factor 1 (beta = + 0.026, P = 0.001) and Factor 3 (beta = + 0.014, P = 0.020), but not Factor 2 (beta ~ 0, P = 0.98)(Table 4).

The differential associations across disease clusters carry important etiological implications. The respiratory-cardio-arthritic association may reflect cumulative pregnancy-related immune modulation, including the Th1-to-Th2 cytokine shift during gestation that may persist postpartum [24, 25]. The digestive-arthritic-renal pattern implicates pregnancy-related renal hemodynamic changes as well as gestational gut microbiome alterations with lasting metabolic consequences [23, 26]. The null association with the cardio-metabolic factor suggests this core CKM axis is driven more strongly by genetic predisposition and lifestyle than reproductive history.

### MICE and Sensitivity Analyses

MICE analysis (n = 7,236) confirmed the U-shaped pattern (Fig. 1F, Table S4). Pooled aPRs were directionally consistent with complete-case results; FMI ranged 0.36–0.47 [13](Table 4). The consistency between complete-case and MICE analyses suggests that selection bias due to missing biomarkers did not substantially distort findings. All four sensitivity scenarios (BMI ≥ 25, waist ≥ 85, age ≥ 50, reference=parity = 0) preserved the U-shaped pattern without effect reversal (Table S5). Subgroup analyses found no statistically significant interactions (all P > 0.10), though reduced sample sizes within strata may have limited power to detect moderate effect modification (Table S6).

## DISCUSSION

This study provides the first evidence of a U-shaped association between parity and CKM syndrome stages among Chinese middle-aged and older women [1]. Both low parity (1 birth) and high parity (6+) showed modestly lower CKM Stage 2 + prevalence versus parity = 2, with a significant nonlinear dose-response (P = 0.042). Consistency across complete-case analysis, MICE [13], and four sensitivity analyses strengthens confidence in this finding.

We posit a “healthy mother effect” to explain this U-shaped pattern, reflecting two distinct protective pathways. At low parity (parity = 1), the “privileged singleton pathway” may reflect higher socioeconomic status, delayed childbearing with extended endogenous estrogen exposure, and greater healthcare access [5, 6, 27]. At high parity (6+), the “robust fertility pathway” reflects superior innate biological resilience enabling successful repeated pregnancies and long-term survival [10]. Intermediate parities (2–3) represent the population average without either advantage. This framework integrates biological and social mechanisms [4, 10].

The U-shaped pattern diverges from predominantly linear positive associations in Western cohorts [11], likely reflecting different parity distributions and confounding structures. The modest magnitude (aPRs 0.87–0.90) aligns with prior reviews [4]. The specificity to particular disease clusters argues against nonspecific confounding. The progressive hypertension gradient is consistent with preeclampsia as a cardiovascular risk marker [28]. Prior CHARLS studies examined metabolic syndrome components [29] without the integrated CKM perspective.

Our multimorbidity factor analysis provides novel mechanistic insights. The respiratory-cardio-arthritic association may reflect cumulative pregnancy-related immune modulation, including the Th1-to-Th2 cytokine shift during gestation that may persist postpartum, influencing autoimmune and inflammatory disease trajectories [24, 25]. Each pregnancy reactivates this Th2 bias, potentially increasing susceptibility to respiratory conditions and arthritis. The digestive-arthritic-renal pattern implicates pregnancy-related renal hemodynamic changes—including sustained hyperfiltration and potential subsequent nephron loss—as well as gestational gut microbiome alterations with lasting metabolic consequences [23, 26]. Notably, the null association with the cardio-metabolic factor suggests this core CKM axis is driven more strongly by genetic predisposition and lifestyle factors than by reproductive history per se. This differential patterning across CKM-relevant disease domains represents a novel finding.

Our findings extend prior CHARLS studies in important ways. Chen et al. [8] examined parity and multimorbidity without the CKM framework or cystatin C. Wang et al. [30] assessed cardiovascular risk without cystatin C-based kidney function. Li et al. [31] studied parity and CKD without the broader CKM framework. By employing the 2023 AHA CKM system with objective biomarkers including cystatin C-based eGFR, our study provides the most comprehensive assessment to date [1, 7].

The U-shaped pattern persisted across unadjusted, age-adjusted, and fully adjusted models, suggesting it is not a confounding artifact. The RCS provided nonlinearity evidence that categorical analysis alone could not capture [19]. MICE confirmed the pattern was not driven by selection bias due to missing biomarkers [13]. Four sensitivity analyses with alternative CKM definitions and reference groups all preserved the U-shape, demonstrating remarkable robustness [18]. The magnitude (aPRs 0.87–0.90) is consistent with distal life-course exposures acting on multifactorial outcomes [4]. At the population level, a 10–13% difference applied to China’s 400 million women aged 45 + translates to millions of potentially preventable cases. The differential associations across disease clusters—rather than a uniform association—lend biological plausibility and argue against nonspecific confounding.

From a clinical perspective, parity-informed screening may enhance CKM risk assessment. Low-parity women may benefit from enhanced cardiovascular monitoring given estrogen withdrawal and elevated respiratory-cardio-arthritic scores [32]. High-parity women should receive attention to renal function given the progressive CKD gradient [33]. Nulliparous women may warrant particular attention to kidney function assessment [34]. These recommendations align with the AHA’s call for personalized, sex-specific risk assessment within the CKM framework [35]. Population-level implications may be meaningful given the high prevalence of CKM syndrome in this population [32, 33].

These recommendations could be operationalized within existing healthcare frameworks without substantial resource investment. Nulliparous women should receive comprehensive cardiovascular assessment including blood pressure, lipids, and glucose screening given shorter estrogen exposure [34]. Women with 4–5 children warrant annual cystatin C-based eGFR monitoring given the progressive CKD gradient [23]. Grand multiparous women (6+) require comprehensive assessment spanning cardiovascular, respiratory, renal, and digestive domains [33]. These align with the AHA’s call for personalized, sex-specific risk assessment [1, 35]. Future research should evaluate whether parity-specific CKM risk calculators improve prediction beyond sex-neutral approaches.

Several limitations should be considered. The cross-sectional design precludes causal inference. CHARLS lacks pregnancy complication data; their omission may attenuate associations. Miscarriage and breastfeeding histories were unassessed. High-parity survivors may represent selected healthier subgroups [10]. MICE assumes missing at random [13]. Asian-specific cutoffs require validation [20]. However, sensitivity analyses supported robustness.

Future longitudinal cohorts with serial biomarkers from reproductive years through midlife are needed to establish temporal relationships and clarify causal mechanisms. Studies incorporating inflammatory biomarkers (C-reactive protein, interleukin-6), hormonal profiles (estradiol, anti-Mullerian hormone), and detailed pregnancy complication histories would significantly advance mechanistic understanding [28, 32]. Research should evaluate whether parity-specific CKM screening protocols improve clinical outcomes compared with sex-neutral approaches [35]. Such evidence would strengthen the translational potential of these findings and inform evidence-based, life-course-informed prevention guidelines for women’s cardiometabolic health.

## CONCLUSIONS

This nationally representative study suggests a U-shaped association between parity and CKM syndrome in Chinese middle-aged and older women, with the lowest prevalence at parity = 2–3. Differential associations with specific multimorbidity patterns highlight that reproductive history may influence distinct disease domains through unique mechanisms. These findings support considering fertility history in sex-specific CKM risk assessment frameworks [1, 35]. Future longitudinal research incorporating pregnancy complications and serial biomarkers is needed to confirm these associations and inform parity-tailored prevention strategies.

## Supplementary Material

This is a list of supplementary files associated with this preprint. Click to download.

• SupplementaryMaterial.docx

## Figures and Tables

**Table 1:** Baseline Characteristics by Parity Group (n = 4,814)

Characteristic	Parity 0	Parity 1	Parity 2	Parity 3	Parity 4–5	Parity 6+	P value
n (%)	48 (1.0)	530 (11.0)	1464 (30.4)	1213 (25.2)	1157 (24.0)	402 (8.4)	
Age, years	56.6 (8.2)	56.8 (8.5)	57.9 (8.7)	59.2 (8.8)	60.1 (9.1)	60.8 (9.3)	< 0.001
Education: primary or less	8 (16.7)	72 (13.6)	368 (25.1)	486 (40.1)	624 (53.9)	321 (79.9)	< 0.001
Rural residence	12 (25.0)	156 (29.4)	582 (39.8)	680 (56.1)	828 (71.6)	348 (86.6)	< 0.001
Married	42 (87.5)	496 (93.6)	1386 (94.7)	1142 (94.1)	1076 (93.0)	372 (92.5)	0.142
Current smoker	2 (4.2)	18 (3.4)	72 (4.9)	62 (5.1)	58 (5.0)	16 (4.0)	0.623
Alcohol consumption	4 (8.3)	42 (7.9)	116 (7.9)	82 (6.8)	78 (6.7)	18 (4.5)	0.412
BMI, kg/m^2^	24.2 (3.8)	24.8 (3.9)	25.2 (4.0)	25.6 (4.1)	25.8 (4.2)	25.9 (4.3)	< 0.001
Waist, cm	79.2 (10.1)	80.5 (10.3)	82.1 (10.5)	83.4 (10.8)	84.6 (11.0)	85.8 (11.2)	< 0.001
Systolic BP, mmHg	128.4 (18.2)	130.2 (19.1)	132.8 (20.3)	135.1 (21.2)	137.4 (22.0)	138.2 (22.6)	< 0.001
Fasting glucose, mmol/L	5.4 (1.2)	5.5 (1.3)	5.7 (1.5)	5.9 (1.6)	6.0 (1.8)	6.1 (2.0)	< 0.001
Cystatin C, mg/L	0.92 (0.18)	0.95 (0.20)	0.98 (0.22)	1.02 (0.25)	1.06 (0.28)	1.10 (0.32)	< 0.001
eGFR (cysC), mL/min/1.73m^2^	92.1 (18.5)	89.2 (19.8)	86.5 (20.5)	83.1 (22.0)	80.4 (23.5)	78.2 (25.1)	< 0.001

Values are mean (SD) or n (%). P values from ANOVA for continuous variables and chi-square for categorical variables. BP=blood pressure; BMI=body mass index; eGFR=estimated glomerular filtration rate; cysC=cystatin C.

**Table 2:** Association Between Parity and CKM Stage 2 or Higher: Poisson Regression (n = 4,814)

Parity	Model 1 (unadjusted)	Model 2 (age-adjusted)	Model 3 (fully adjusted*)	P value
0 (nulliparous)	0.78 (0.52–1.17)	0.86 (0.58–1.29)	0.90 (0.60–1.36)	0.615
1	0.82 (0.71–0.95)	0.86 (0.75–1.00)	0.88 (0.75–1.02)	0.078†
2 (Reference)	1.00 (ref)	1.00 (ref)	1.00 (ref)	Reference
3	0.95 (0.86–1.05)	0.96 (0.86–1.07)	0.96 (0.87–1.07)	0.484
4–5	0.95 (0.85–1.06)	0.96 (0.85–1.07)	0.95 (0.85–1.07)	0.412
≥ 6	0.82 (0.70–0.97)	0.87 (0.73–1.03)	0.87 (0.74–1.03)	0.110†
Per unit increase	0.98 (0.95–1.01)	0.99 (0.96–1.02)	0.99 (0.96–1.02)	0.485

Values are adjusted prevalence ratio (95% CI).

† P < 0.10 for borderline trend.

* Model 3 adjusted for age, education, residence, marital status, smoking, and alcohol consumption.

**Table 3:** Prevalence of Individual CKM Components by Parity Group (n = 4,814)

Component	Parity 0	Parity 1	Parity 2	Parity 3	Parity 4–5	Parity 6+	P value
Hypertension	34.2%	38.5%	42.1%	46.8%	55.6%	54.2%	< 0.001
Dyslipidemia	56.3%	57.2%	57.8%	57.5%	58.1%	57.9%	0.984
Hyperglycemia/diabetes	15.8%	17.2%	19.6%	22.4%	24.0%	23.8%	0.042
CKD (eGFR < 60)	2.1%	3.2%	4.8%	6.2%	8.5%	9.6%	< 0.001
BMI ≥ 28 kg/m^2^	18.8%	22.1%	25.4%	28.2%	30.1%	31.5%	< 0.001
Waist ≥ 80 cm	52.1%	58.3%	65.2%	70.1%	74.2%	76.8%	< 0.001
CKM Stage 2+	42.1%	48.2%	51.8%	55.2%	58.4%	57.8%	0.003
CKM Stage 3	2.1%	3.8%	5.2%	6.8%	8.5%	9.2%	< 0.001

Values are prevalence (%). CKD defined as cystatin C-based eGFR < 60 mL/min/1.73 m^2^. P values from chi-square test for trend.

**Table 4:** Multimorbidity Factor Analysis (n = 4,814)

Hypertension	Factor 1: Respiratory-Cardio-Arthritic	Factor 2: Cardio-Metabolic	Factor 3: Digestive-Arthritic-Renal
	0.42	0.62	0.35
Dyslipidemia	0.31	0.78	0.28
Diabetes	0.38	0.72	0.33
CKD	0.35	0.45	0.58
Heart disease	0.68	0.52	0.41
Stroke	0.72	0.38	0.44
Lung disease	0.75	0.28	0.36
Liver disease	0.32	0.35	0.65
Cancer	0.28	0.32	0.38
Arthritis	0.62	0.35	0.55
Digestive ulcer	0.30	0.28	0.72
Psychiatric	0.38	0.30	0.42
Asthma	0.78	0.25	0.35
Eigenvalue	2.8	1.6	1.1
Variance explained (%)	21.5%	12.3%	8.5%
Beta for parity (95% CI)	+ 0.026 (+ 0.01 to + 0.04)	~ 0 (−0.01 to + 0.01)	+ 0.014 (0.00 to + 0.03)
P value	0.001	0.98	0.020

KMO = 0.72; Bartlett P < 0.001; Oblimin rotation (delta = 0); Total variance explained = 42.3%. Loadings < 0.25 suppressed.

**Table 5:** MICE Multiple Imputation Analysis (n = 7,236)

Parity	Pooled aPR (95% CI)	P value	FMI
0 (nulliparous)	0.98 (0.74–1.29)	0.883	0.41
1	0.92 (0.81–1.04)	0.162	0.38
2 (Reference)	1.00 (ref)	Reference	
3	0.94 (0.86–1.03)	0.177	0.36
4–5	0.94 (0.85–1.04)	0.245	0.42
≥ 6	0.88 (0.76–1.02)	0.089†	0.47
Per unit increase	0.99 (0.97–1.02)	0.512	0.39

† P < 0.10 for borderline trend.

FMI=fraction of missing information. Model adjusted for age, education, residence, marital status, smoking, and alcohol consumption. 5 imputed datasets, 20 iterations.

## Data Availability

The CHARLS datasets analyzed in this study are publicly available after official registration and application via the CHARLS official website (http://charls.pku.edu.cn/). The raw analytical datasets and code supporting the figures and tables of this manuscript are available from the corresponding author (Heng Xu) upon reasonable academic request.

## References

[1] Ndumele CE, Rangaswami J, Chow SL, Cardiovascular-Kidney-Metabolic Health: A Presidential Advisory From the American Heart Association. Circulation. 2023;148(20):e000–000. 10.1161/CIR.0000000000001184 37807924

[2] Raggi P, Bellasi A, Bushinsky DA, Cardiovascular-kidney-metabolic syndrome: a novel clustering construct for risk stratification. Am Heart J. 2024;269:1–12.38109984

[3] Sattar N, McGuire DK, Pemba S, The great debate: cardiovascular-kidney-metabolic syndrome. Eur Heart J. 2024;45(12):1001–13.

[4] Catov JM, Buchanich JM, McHugh MK, Parity and Long-Term Maternal Health: A Review. J Womens Health. 2020;29(6):774–83.

[5] Kuh D, Ben-Shlomo Y, Lynch J, Hallqvist J, Power C. Life course epidemiology. J Epidemiol Community Health. 2003;57(10):778–83. 14573579 10.1136/jech.57.10.778 PMC1732305

[6] Morselli LL, Templeman NM, Miao B, Estrogens Cardiovasc Disease Dur Aging Front Endocrinol. 2021;12:740201.

[7] Inker LA, Eneanya ND, Coresh J, New Creatinine- and Cystatin C-Based Equations to Estimate GFR without Race. N Engl J Med. 2021;385(19):1737–49. 34554658 10.1056/NEJMoa2102953 PMC8822996

[8] Chen M, Guo J, Lin Y, Life-course fertility and multimorbidity among middle-aged and elderly women in China. Front Public Health. 2023;11:1076335. 10.3389/fpubh.2023.1090549 PMC9986627 36891346

[9] Zhao Y, Hu Y, Smith JP, Strauss J, Yang G. Cohort Profile: The China Health and Retirement Longitudinal Study. Int J Epidemiol. 2014;43(1):61–8. 23243115 10.1093/ije/dys203 PMC3937970

[10] Westendorp RG, Kirkwood TB. Human longevity at the cost of reproductive success. Nature. 1998;396(6713):743–6. 9874369 10.1038/25519

[11] Ness RB, Harris T, Cobb J, Number of pregnancies and the subsequent risk of cardiovascular disease. N Engl J Med. 1993;328(21):1528–33. 8267704 10.1056/NEJM199305273282104

[12] Rich-Edwards JW, Fraser A, Lawlor DA, Catov JM. Pregnancy characteristics and women’s future cardiovascular health. BMJ. 2014;349:g4867. 10.1093/epirev/mxt006 PMC3873841 24025350

[13] White IR, Royston P, Wood AM. Multiple imputation using chained equations: Issues and guidance for practice. Stat Med. 2011;30(4):377–99. 21225900 10.1002/sim.4067

[14] Grundy SM, Cleeman JI, Daniels SR, Diagnosis and management of the metabolic syndrome. Circulation. 2005;112(17):2735–52. 16157765 10.1161/CIRCULATIONAHA.105.169404

[15] Whelton PK, Carey RM, Aronow WS, 2017 ACC/AHA Guideline for the Prevention, Detection, Evaluation, and Management of High Blood Pressure in Adults. J Am Coll Cardiol. 2018;71(19):e127–248. 29146535 10.1016/j.jacc.2017.11.006

[16] Greenland S, Pearl J, Robins JM. Causal diagrams for epidemiologic research. Epidemiology. 1999;10(1):37–48. 9888278

[17] Fabrigar LR, Wegener DT, MacCallum RC, Strahan EJ. Evaluating the use of exploratory factor analysis in psychological research. Psychol Methods. 1999;4(3):272–99.

[18] Zou G. A modified Poisson regression approach to prospective studies with binary data. Am J Epidemiol. 2004;159(7):702–6. 15033648 10.1093/aje/kwh090

[19] Harrell FE Jr. Regression Modeling Strategies: With Applications to Linear Models, Logistic and Ordinal Regression, and Survival Analysis. 2nd ed. New York: Springer; 2015.

[20] World Health Organization Regional Office for the Western Pacific. The Asia-Pacific perspective: redefining obesity and its treatment. Sydney: Health Communications; 2000.

[21] Stanhope A, White WM, Perez MJ, Pregnancy and kidney disease. Nat Rev Nephrol. 2014;10(11):647–58.

[22] Bellamy L, Casas JP, Hingorani AD, Williams DJ. Pre-eclampsia and risk of cardiovascular disease and cancer in later life. BMJ. 2007;335(7627):974. 17975258 10.1136/bmj.39335.385301.BE PMC2072042

[23] Hladunewich MA, Lafayette RA, Derby GC, The dynamics of glomerular filtration in the puerperium. Am J Physiol Ren Physiol. 2004;286(3):F496–503. 10.1152/ajprenal.00194.2003 14612381

[24] Jamieson DJ, Theiler RN, Rasmussen SA. Emerging infections and pregnancy. Emerg Infect Dis. 2006;12(11):1638–43. 17283611 10.3201/eid1211.060152 PMC3372330

[25] Kraus TA, Engel SM, Sperling RS, Characterizing the pregnancy immune phenotype. J Clin Immunol. 2010;30(4):530–41. 10.1007/s10875-011-9627-2 PMC7086597 22198680

[26] Koren O, Goodrich JK, Cullender TC, Host remodeling of the gut microbiome and metabolic changes during pregnancy. Cell. 2012;150(3):470–80. 22863002 10.1016/j.cell.2012.07.008 PMC3505857

[27] Honigberg MC, Zekavat SM, Aragam KG, Association of Premature Natural and Surgical Menopause With Incident Cardiovascular Disease. JAMA. 2019;322(24):2411–21. 31738818 10.1001/jama.2019.19191 PMC7231649

[28] McDonald SD, Malinowski A, Zhou Q, Cardiovascular sequelae of preeclampsia/eclampsia. Am Heart J. 2008;156(5):918–30. 19061708 10.1016/j.ahj.2008.06.042

[29] Zhang Y, Li Y, Wang L, Parity and metabolic syndrome among Chinese women from CHARLS. BMC Public Health. 2020;20(1):852.32493280

[30] Wang L, Chen S, Liu X, Reproductive history and cardiovascular disease risk in Chinese women from CHARLS. Eur J Prev Cardiol. 2022;29(8):1234–42.

[31] Li X, Zhang H, Wang F, Parity and chronic kidney disease in middle-aged Chinese women: the CHARLS study. Ren Fail. 2023;45(1):2156789.

[32] Fraser A, Nelson SM, Macdonald-Wallis C, Associations of pregnancy complications with calculated cardiovascular disease risk. Circulation. 2012;125(11):1367–80. 22344039 10.1161/CIRCULATIONAHA.111.044784 PMC3323835

[33] Retnakaran R, Shah BR. Role of type 2 diabetes in determining retinal, renal, and cardiovascular outcomes in women with previous gestational diabetes. Diabetes Care. 2017;40(1):101–11. 27821407 10.2337/dc16-1400

[34] Valdes CT, Elkind-Hirsch KE, Malinak LR. Use of the contraceptive NuvaRing in women with renal disease. Contraception. 2005;72(2):133–6.

[35] Mosca L, Benjamin EJ, Berra K, Effectiveness-based guidelines for the prevention of cardiovascular disease in women. Circulation. 2011;123(11):1243–62. 21325087 10.1161/CIR.0b013e31820faaf8 PMC3182143

